# Supplementary material for: Comparative genomics provides new insights into the diversity, physiology, and sexuality of the only industrially exploited tremellomycete: Phaffia rhodozyma
Source: BMC Genomics. 2016 Nov 9;17:901. doi: 10.1186/s12864-016-3244-7 (PMC5103461; doi:10.1186/s12864-016-3244-7)
Supplement: Additional file 6: — List of orphan genes with links to PFAM (related to Additional file 1: Table S1). (ZIP 1428 kb) [file 12864_2016_3244_MOESM6_ESM.zip › BLAST_HTML_FTR/G01663_P.html]

BLAST Search Results


```
BLASTP 2.2.27+


Reference:
Stephen F. Altschul, Thomas L. Madden, Alejandro A. Schäffer,
Jinghui Zhang, Zheng Zhang, Webb Miller, and David J. Lipman (1997),
"Gapped BLAST and PSI-BLAST: a new generation of protein database
search programs", Nucleic Acids Res. 25:3389-3402.


Reference for
composition-based statistics:
Alejandro A. Schäffer, L. Aravind, Thomas L. Madden, Sergei
Shavirin, John L. Spouge, Yuri I. Wolf, Eugene V. Koonin, and
Stephen F. Altschul (2001), "Improving the accuracy of PSI-BLAST
protein database searches with composition-based statistics and
other refinements", Nucleic Acids Res. 29:2994-3005.


Database: nr
           71,551,133 sequences; 26,053,659,533 total letters


Query= G01663_P

Length=425
                                                                      Score     E
Sequences producing significant alignments:                          (Bits)  Value

emb|CDZ97249.1|  hypothetical protein [Xanthophyllomyces dendrorh...   436    8e-148


 >emb|CDZ97249.1| hypothetical protein [Xanthophyllomyces dendrorhous]
Length=340

 Score =  436 bits (1121),  Expect = 8e-148, Method: Compositional matrix adjust.
 Identities = 243/250 (97%), Positives = 243/250 (97%), Gaps = 7/250 (3%)

Query  182  ALVSGTNPPYSVDSSDRLDENFSTMNPLFIRRSASDPVSPASPPYASSSLASLPSGAAPA  241
            ALVSGTNPPYSVDSSDRLDENFSTMNPLFIRRSASDPVSPASPPYASSSLASLPSGAAPA
Sbjct  91   ALVSGTNPPYSVDSSDRLDENFSTMNPLFIRRSASDPVSPASPPYASSSLASLPSGAAPA  150

Query  242  WKAARDAIASRGQSDERQMDSALPPPPLGKPISSNWVVLKTTSPTLSHSTSLFFHPTSAS  301
            WKAARDAIASRGQSDERQMDSALPPPPLGKPISSNWVVLKTTSPTLSHSTSLFFHPTSAS
Sbjct  151  WKAARDAIASRGQSDERQMDSALPPPPLGKPISSNWVVLKTTSPTLSHSTSLFFHPTSAS  210

Query  302  TNSSPTSSLRESDGGSPISLKNSVRKKLSALS-------SRSNTATSYTRKTSTNHIRRN  354
            TNSSPTSSLRESDGGSPISLKNSVRKKLSALS       SRSNTATSYTRKTSTNHIRRN
Sbjct  211  TNSSPTSSLRESDGGSPISLKNSVRKKLSALSVRKASDESRSNTATSYTRKTSTNHIRRN  270

Query  355  ILSGRQKDPSASSNRDSGDSLEQEYFSSSSSSTSSLEIEFLTRVTTPGCEQKGRHQSVET  414
            ILSGRQKDPSASSNRDSGDSLEQEYFSSSSSSTSSLEIEFLTRVTTPGCEQKGRHQSVET
Sbjct  271  ILSGRQKDPSASSNRDSGDSLEQEYFSSSSSSTSSLEIEFLTRVTTPGCEQKGRHQSVET  330

Query  415  LLPSRIFLAL  424
            LLPSRIFLAL
Sbjct  331  LLPSRIFLAL  340


 Score =  144 bits (363),  Expect = 2e-35, Method: Compositional matrix adjust.
 Identities = 90/91 (99%), Positives = 90/91 (99%), Gaps = 0/91 (0%)

Query  1   MRLLLGKKSNRSLKQATSSSPPPPPSPLTYPLPVLPPLVHVQTASCPTSPGKPTPPLGKS  60
           MRLLLGKKSNRSLKQATSSSPPPPPSPLTYPLPVLPPLVHVQTASCPTSPGKPTPPLGKS
Sbjct  1   MRLLLGKKSNRSLKQATSSSPPPPPSPLTYPLPVLPPLVHVQTASCPTSPGKPTPPLGKS  60

Query  61  SVTTPLRIHAMFSRSFSMNDVSLTGHPGQPV  91
           SVTTPLRIHAMFSRSFSMNDVSLTGHPGQP 
Sbjct  61  SVTTPLRIHAMFSRSFSMNDVSLTGHPGQPA  91


Lambda      K        H        a         alpha
   0.310    0.124    0.358    0.792     4.96 

Gapped
Lambda      K        H        a         alpha    sigma
   0.267   0.0410    0.140     1.90     42.6     43.6 

Effective search space used: 4108923442048


  Database: nr
    Posted date:  Sep 23, 2015 12:05 AM
  Number of letters in database: 26,053,659,533
  Number of sequences in database:  71,551,133


Matrix: BLOSUM62
Gap Penalties: Existence: 11, Extension: 1
Neighboring words threshold: 11
Window for multiple hits: 40
```
